# Letter to the Editor: Ultrasound findings for the diagnosis of biliary atresia in neonates

**DOI:** 10.1590/0100-3984.2025.0116

**Published:** 2026-09-08

**Authors:** Kaushik Sankaramoorthy, Harshavardhan Balaganesan, Sai Shankar Manakuzhy Gopalakrishnan, Remya R. Nair

**Affiliations:** 1 Shri Sathya Sai Medical College & Research Institute, affiliated with Sri Balaji Vidyapeeth, Puducherry, India

## Dear Editor,

We read with interest the excellent article, authored by Di Puglia et al.^**(^1^)**^, regarding the most relevant ultra-sonographic features in biliary atresia (BA). The authors are to be commended for their diligent assessment of gallbladder morphology, triangular cord (TC) thickening, hepatic artery enlargement, and ancillary Doppler find-ings, all of which reinforce the role of ultrasound as the first line of diagnostic imaging in neonatal cholestasis. The fact that gallbladder disease and TC thickening both occurred in nearly all subjects supports the diagnostic role of these sonographic markers.

This study stands out because of the systematic ap-proach, uniform fasting protocol, and specialty image reporting. The documentation of less well-documented appearances such as porta hepatis cysts, subcapsular arterial supply, and fetal remnant biliary structures ex-pands the described imaging range of BA provided by the authors and highlights the signs with prognostic and surgical significance.

It should also be pointed out that, although hepato-biliary scintigraphy continues to be an adjunctive exami-nation in neonatal cholestasis evaluation, it is relatively nonspecific. Data in the current literature^**(^2^,^3^)**^ attests to the fact that failure of excretion of the tracer beyond the liver is a feature of BA but can be encountered in advanced neonatal hepatitis as well. However, the lack of gallbladder visualization on ultrasound, together with a lack of tracer excretion on hepatobiliary scintigraphy, increases the diagnostic certainty considerably. Other than the strengths noted by the authors, we would like to suggest a few for future reference. First, definitive objective diagnostic cut-offs for gallbladder length (≤ 1.5 cm) and TC thickness (> 2 mm) would create the foundation of a reliable multicenter sonographic scor-ing system. Measurement of interobserver variability, as investigated in previous studies with the use of pediatric shear-wave elastography^**(^4^)**^, would further enhance reli-ability. In addition, the fact that the authors employed only standard and Doppler ultrasound shows that it is an efficient, cost-effective diagnostic approach that could be used in resource-constrained settings where magnetic resonance cholangiopancreatography and hepatobiliary scintigraphy might not be available.

In summary, the Di Puglia et al.^**(^1^)**^ study provides considerable support of the evidence in favor of ultrasound as a basis for assessing neonatal cholestasis and makes a significant contribution to the enhancement of early BA detection and timely surgical referral.
